# Correlation Between Dual-Time-Point FDG PET and Tumor Microenvironment Immune Types in Non-Small Cell Lung Cancer

**DOI:** 10.3389/fonc.2021.559623

**Published:** 2021-03-18

**Authors:** Jianyuan Zhou, Sijuan Zou, Siyuan Cheng, Dong Kuang, Dan Li, Lixing Chen, Cong Liu, Jianhua Yan, Xiaohua Zhu

**Affiliations:** ^1^ Department of Nuclear Medicine and PET, Tongji Hospital, Tongji Medical College, Huazhong University of Science and Technology, Wuhan, China; ^2^ Department of Pathology, Tongji Hospital, Tongji Medical College, Huazhong University of Science and Technology, Wuhan, China; ^3^ Shanghai Key Laboratory of Molecular Imaging, Shanghai University of Medicine and Health Sciences, Shanghai, China

**Keywords:** DTP ^18^F-FDG PET, PD-L1, tumor microenvironment immune types, NSCLC, metabolic parameters

## Abstract

**Purpose:**

Dual-time-point ^18^F-fluorodeoxyglucose positron emission tomography (DTP ^18^F-FDG PET), which reflects the dynamics of tumor glucose metabolism, may also provide a novel approach to the characterization of both cancer cells and immune cells within the tumor immune microenvironment (TIME). We investigated the correlations between the metabolic parameters (MPs) of DTP ^18^F-FDG PET images and the tumor microenvironment immune types (TMITs) in patients with non-small cell lung cancer (NSCLC).

**Methods:**

A retrospective analysis was performed in 91 patients with NSCLC who underwent preoperative DTP ^18^F-FDG PET/CT scans. MPs in the early scan (eSUVmax, eSUVmean, eMTV, eTLG) and delayed scan (dSUVmax, dSUVmean, dMTV, dTLG) were calculated, respectively. The change in MPs (ΔSUVmax, ΔSUVmean, ΔMTV, ΔTLG) between the two time points were calculated. Tumor specimens were analyzed by immunohistochemistry for PD-1/PD-L1 expression and CD8^+^ tumor-infiltrating lymphocytes (TILs). TIME was classified into four immune types (TMIT I ~ IV) according to the expression of PD-L1 and CD8^+^ TILs. Correlations between MPs with TMITs and the immune-related biomarkers were analyzed. A composite metabolic signature (Meta-Sig) and a combined model of Meta-Sig and clinical factors were constructed to predict patients with TMIT I tumors.

**Results:**

eSUVmax, eSUVmean, dSUVmax, dSUVmean, ΔSUVmax, ΔSUVmean, and ΔTLG were significantly higher in PD-L1 positive patients (*p* = 0.0007, 0.0006, < 0.0001, < 0.0001, 0.0002, 0.0002, 0.0247, respectively), and in TMIT-I tumors (*p* = 0.0001, < 0.0001, < 0.0001, < 0.0001, 0.0009, 0.0009, 0.0144, respectively). Compared to stand-alone MP, the Meta-Sig and combined model displayed better performance for assessing TMIT-I tumors (Meta-sig: AUC = 0.818, sensitivity = 86.36%, specificity = 73.91%; Model: AUC = 0.869, sensitivity = 77.27%, specificity = 82.61%).

**Conclusion:**

High glucose metabolism on DTP ^18^F-FDG PET correlated with the TMIT-I tumors, and the Meta-Sig and combined model based on clinical and metabolic information could improve the performance of identifying the patients who may respond to immunotherapy.

## Introduction

Lung cancer is the leading cause of cancer death in China (1). Non-small cell lung cancer (NSCLC) accounts for more than 80% of all lung cancer cases. Over the last decade, the immune checkpoint inhibitors (ICIs) targeting the programmed death protein 1 (PD-1)/programmed death ligand 1 (PD-L1) axis have shown significant clinical benefit for advanced NSCLC patients. The expression of PD-L1 on tumor cells is considered as a predictive biomarker for the response to anti-PD-1/PD-L1 ICIs (2). However, not all patients with positive PD-L1 expression respond well to immunotherapy. It suggests that other tumor immune microenvironment (TIME) factors may also affect the response to the ICIs (3). In addition to PD-L1 expression, CD8^+^ tumor-infiltrating lymphocytes (TILs) might play an important role in anti–PD-1/PD-L1 therapies (2). Without CD8^+^ TILs, it’s unlikely that blocking PD-1 or PD-L1 causes any tumor inhibition (4). Characterized by high infiltration of CD8^+^ cytotoxic lymphocytes, the infiltrated–inflamed TIME has significantly better responses to ICIs (5). Therefore, it was proposed that TIME could be classified into four subtypes based on PD-L1 and CD8^+^ TILs status (4). Tumors with high PD-L1 expression and the presence of CD8^+^ TILs are classified as tumor microenvironment immune type I (TMIT-I), a immunologically ‘hot’ subtype that would likely benefit from anti-PD-1/PD-L1 therapies (6). However, there is no noninvasive method to identify TMIT I tumors, and up to now the overall and dynamic detection of TIME biomarkers is still challenging.

Among the image-based modalities for non-invasive tumor assessment, ^18^F-fluorodeoxyglucose positron emission tomography/computed tomography (^18^F-FDG PET/CT) is the most common one of patients with NSCLC (7). Glucose metabolism is closely related to the characteristics of TIME. As a nutrient, glucose is actively entrapped in neoplastic tissue and tumor-related activated immune cells (8). Previous studies have established the correlation between the MPs [maximum and mean standard uptake value (SUVmax and SUVmean)] of ^18^F-FDG PET and the expression of immune markers (PD-1, PD-L1 and CD8) in patients with NSCLC (9–16). However, little attention was paid to CD8^+^ TILs and tumor immune types. The predictive value of tumor metabolism solely based on SUVmax remains weak in patients undergoing ICI immunotherapy. Dual-time-point (DTP) ^18^F-FDG PET, which reflects the dynamics of glucose metabolism, is expected to be a potential imaging method to reveal the TIME information. Up to date, the correlation between metabolic parameters (MPs) on DTP FDG PET and TMITs in pretreated NSCLC remains unclear.

This retrospective study was conducted to correlate a number of MPs of DTP ^18^F-FDG PET with immune markers and TMITs in a cohort of pretreated NSCLC patients. We hypothesize that the abundant metabolic information on DTP FDG PET imaging defines the TMITs of NSCLC and helps optimize patient selection for ICIs treatment.

## Materials and Methods

### Patient Population

Patients who underwent pretreatment DTP ^18^F-FDG PET/CT scans in Tongji hospital for NSCLC diagnosis and staging from December 2014 to December 2017, were retrospectively reviewed. Eligible patients were histologically confirmed with NSCLC, underwent initial PET scan less than 30 days from surgery (or biopsy), with tumor size ≥ 1 cm in diameter. Key exclusion criteria were: patients that received anti-tumor therapy before surgery (or biopsy), and patients whose tumor specimens were not available for immunohistochemistry. This retrospective study was approved by the institutional review board.

### DTP ^18^F-FDG PET/CT Acquisition Protocol and Image Analysis

In each patient, 3.7 MBq/kg FDG was intravenously administered after fasting for at least 6 h. The blood glucose concentration was lower than 200 mg/dL before injecting. PET/CT images were obtained by a PET/CT scanner (Discovery 690 PET/CT, GE) at approximately 60 ± 5 min (early) and 120 ± 5 min (delayed) after FDG administration. Whole body images were obtained from the base of the skull to mid-thigh by means of an integrated PET/CT tomography (5 to 7 bed positions with 3 min per bed position). PET images were attenuation-corrected and anatomically fused with low-dose CT images, and reconstructed onto a 128 × 128 matrix. A low-dose helical CT scan (120 kV, 120 mA, slice thickness, 3.75mm) was performed for anatomical correlation and attenuation correction.

Images were analyzed by two board-certified nuclear medicine physicians. Tumor mass area of increased radiotracer uptake was first identified, then a semi-automated, ellipsoid 3D-isocontour volume of interest (VOI) with threshold of 40%SUVmax was marked around the tumor for the measurement of SUVmax, SUVmean and metabolic tumor volume (MTV). For tumors with low uptake, VOI was obtained by manually delineating the boundary layer by layer along the tumors, then SUVs and MTV (with threshold of 40%SUVmax) were automatically calculated within each VOI. Total lesion glycolysis (TLG) and the change of SUVmax, SUVmean, MTV and TLG were calculated according to the following formula: TLG = SUVmean × MTV, ΔMP = dMP – eMP.

### Immunohistochemistry Analysis

Immunohistochemistry was performed using 4 µm sections from a paraffin-embedded tissue block as previously described (17). Briefly, the sections were deparaffinized in xylene and rehydrated in graded ethanol and distilled water. Slides were auto-stained with primary antibodies raised against CD8^+^ (ZA-0508, ZSGB-BIO, China), PD-1 (Abcam, EPR4877(2), ab137132), PD-L1 (ZA-0629, ZSGB-BIO, China). The PD-L1 immunostaining results were classified into two groups based on staining intensity and proportion of tumor cell positivity (18). Staining intensity was scored as follows: 0, negative staining; 1, weak staining; 2, moderate staining; and 3, strong staining (more intense than alveolar macrophages). Case in which more than 5% of tumor cells displayed a staining intensity ≥2 was considered positive. Case with staining intensity <2 or less than 5% of tumor cells was defined negative. The expressions of PD-1 and CD8^+^ TIL were evaluated according to the average number of positively stained cells in 3 randomly selected high-power fields in each case. The numbers of CD8^+^ TILs were classified into two groups based on the median value: CD8^+^ TILs^+^ (n ≤100), CD8^+^ TILs^-^ (n> 100).

Four tumor microenvironment immune types was classified as reported (3, 6, 19): TMIT-I (PD-L1^+^, CD8^+^ TILs^+^); TMIT-II (PD-L1^-^, CD8^+^ TILs^-^); TMIT-III (PD-L1^+^, CD8^+^ TILs^-^) and TMIT-IV (PD-L1^-^, CD8^+^ TILs^+^).

### Statistical Analysis

Data was analyzed with the SPSS statistical package, MedCalc and R software. The distribution of variables was checked using Shapiro-Wilk test. For continuous data, the differences between two groups were assessed using Mann-Whitney *U* test or Student’s t-test. Differences among multi-group were compared using one-way analysis of variance (ANOVA) (with least significant difference method) or Kruskal-Wallis H test, when appropriate. Spearman’s correlation coefficients were calculated. The least absolute shrinkage and selection operator (LASSO) algorithm method using 10-fold cross-validation was employed to select the optimal features. Features with non-zero coefficients at the minimum of lambda were selected from the candidate MPs to construct a metabolic signature (Meta-Sig). Multivariate logistic regression analysis with backward stepwise elimination method was performed to construct a combined model, based on clinical factors and the Meta-Sig. The receiver operating characteristic (ROC) curves and DeLong test were used to compare the area under the curves (AUCs) for predicting TMIT I tumors. Furthermore, decision curve analysis (DCA) was used to evaluate the clinical usefulness of the combined model by quantifying the net benefits at different threshold probabilities. *P*<0.05 was considered to be statistically significant.

## Results

### Patient Characteristics

A total of 91 patients (68 with adenocarcinoma, 22 with squamous cell carcinoma and 1 with adenosquamous carcinoma; 47 male, 44 female) were included. The median age of these patients was 59 years (range 36~78). Patients’ demographics and the median value of twelve MPs of DTP ^18^F-FDG PET images were shown in **Table 1**.

**Table 1:** Demographic and clinical data of all patients.

Characteristics		No. (%)
Age	Median (range)	59 (36~78) years
Gender	Male	47 (51.65%)
	Female	44 (48.35%)
Smoking status	Smoker	37 (40.70%)
	Non-smoker	54 (59.30%)
CEA	Median (range)	2.52 (0.50~397.51) ug/L*
CYFRA21	Median (range)	2.49 (0.94~28.07) ng/ml*
SCC Ag	Median (range)	0.8 (0.30~47.99) μg/L*
Histology	SCC	22 (24.18%)
	ADC	68 (74.73%)
	ASC	1 (1.1%)
Pathologic stage	I	37 (40.70%)
	II	22 (24.2%)
	III	23 (25.30%)
	IV	9 (9.90%)
eSUVmax	Median (range)	8.40 (0.9~20.2)
eSUVmean	Median (range)	5.00 (0.6~12.00)
eMTV	Median (range)	5.78 (0.83~96.14) cm^3^
eTLG	Median (range)	24.68 (0.85~663.37) g
dSUVmax	Median (range)	13.10 (0.80~27.90)
dSUVmean	Median (range)	7.60 (0.60~16.00)
dMTV	Median (range)	5.43 (0.61~89.45) cm^3^
dTLG	Median (range)	30.69 (0.79~796.11) g
ΔSUVmax	Median (range)	2.80 (-0.2~8.500)
ΔSUVmean	Median (range)	1.90 (-0.1~4.30)
ΔMTV	Median (range)	-0.65 (-13.34~4.30) cm^3^
ΔTLG	Median (range)	5.75 (-13.88~151.76) g

*Ten patients’ data on tumor markers were absence.

ADC, adenocarcinoma; SCC, squamous cell carcinoma; ASC, adenosquamous carcinoma; CEA, carcinoembryonic antigen; CYFRA21, cytokeratin 19-fragments; SCC Ag, squamous cell carcinoma antigen; SUVmax, maximum standard uptake value; SUVmean, mean standard uptake value; MTV, metabolic tumor volume; TLG, total lesion glycolysis.

Positive PD-L1 immunostaining was observed on the membranes and/or in the cytoplasm of tumor cells. Positive PD-L1 expressions were noted in 33 of the 91 (36.26%) patients. The median value of CD8^+^ TILs and PD-1 TILs was 100 (range 0~300) and 74 (range 0~282) respectively. The percentage of TMIT-I tumors was 24.18% (22/91) in this study.

### Characterization of TMITs

**Figure 1:**
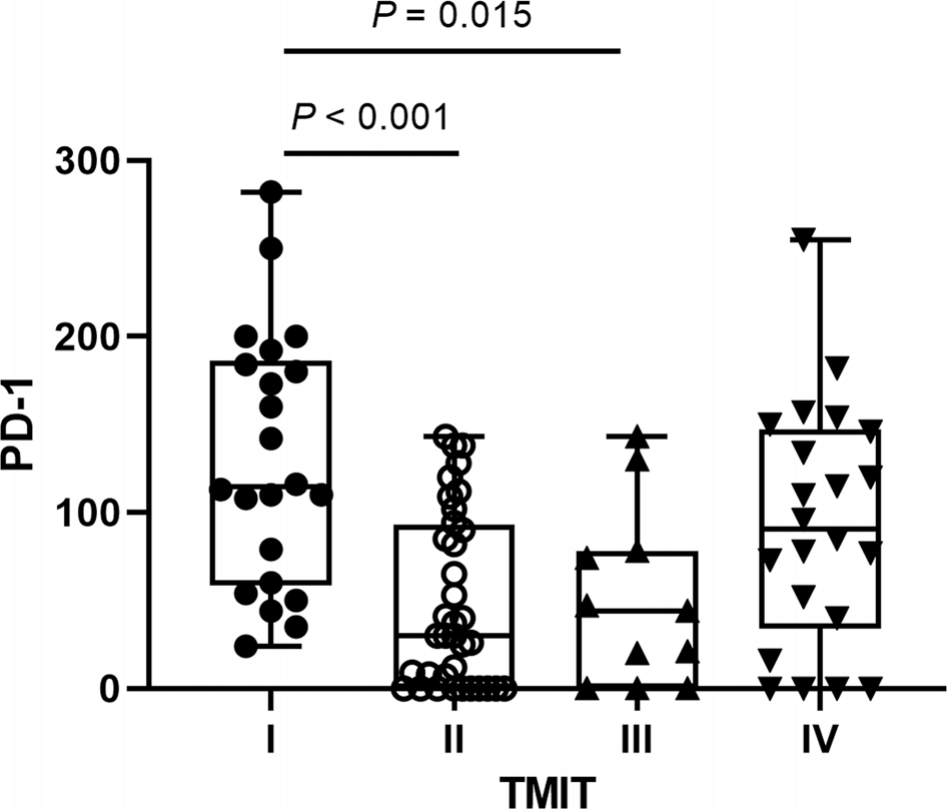
The distributions of programmed cell death 1 (PD‐1) expression according to different tumor microenvironment immune types (TMITs). The PD-1 expression was significantly higher in TMIT I tumors than TMIT II and III tumors.

The percentages of four TMITs were as follows: 22 (24.18%) TMIT I, 36 (39.56%) TMIT II, 11 (12.09%) TMIT III and 22 (24.18%) TMIT IV. PD-1 expression was significantly higher in TMIT I tumors than TMIT II and III tumors (*p* < 0.001, 0.015), but no statistically significant difference was found between TMIT I and IV groups (*p* = 0.584) (**Figure 1**). The spearman’s analysis showed a statistically significant correlation between the PD-1 and CD8^+^ TILs (*rho* = 0.543, *p* < 0.001). PD-1 and CD8^+^ TILs were significantly higher in PD-L1 positive patients (*p* = 0.015 and 0.004, respectively).

### Correlations Between PD-L1 Expression and MPs on DTP FDG PET

**Figure 2:**
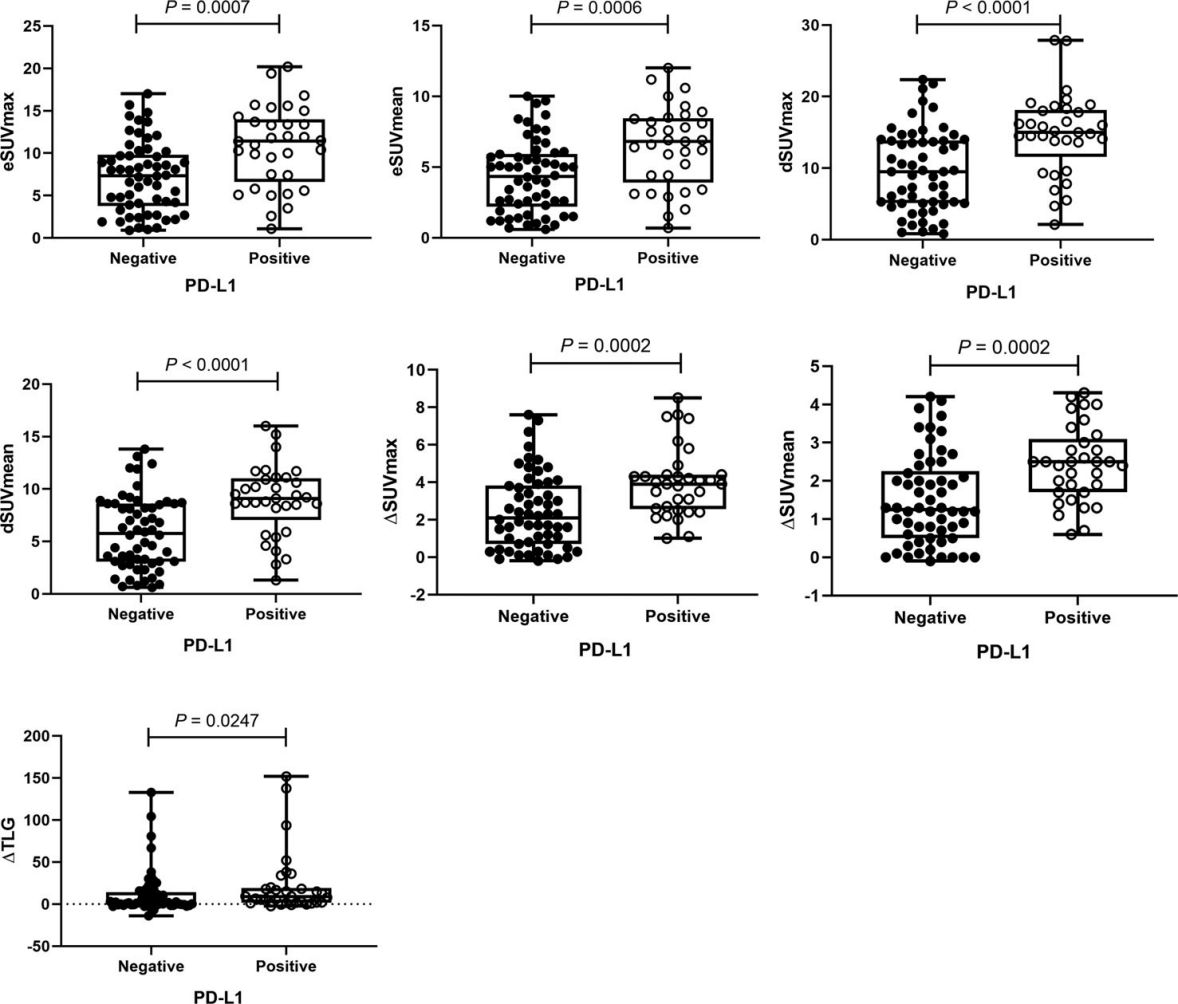
The distributions of metabolic parameters (MPs) according to programmed cell death‐ligand 1 (PD‐L1) protein expression. The MPs were significantly higher in patients with PD‐L1 positivity than those with PD‐L1 negativity.

By Mann-Whitney *U* test, PD-L1 positive patients showed higher early SUVmax (eSUVmax) (*p* = 0.0007), early SUVmean (eSUVmean) (*p* = 0.0006), delayed SUVmax (dSUVmax) (*p* < 0.0001), delayed SUVmean (dSUVmean) (*p*<0.0001), ΔSUVmax (*p* = 0.0002), ΔSUVmean (*p* = 0.0002) and ΔTLG (*p* = 0.0247) in **Figure 2**. ROC curve for MPs showed moderate ability for predicting PD-L1 expression in **Table 2**.

**Table 2:** ROC analysis for the metabolic parameters according to PD-L1 expression.

Parameters	sensitivity	specificity	cutoff	AUC	95%CI
eSUVmax	66.67%	75.86%	9.7	0.714	0.610 - 0.804
eSUVmean	63.64%	77.59%	6.0	0.716	0.612 - 0.806
dSUVmax	66.67%	79.31%	14.0	0.750	0.648 - 0.835
dSUVmean	72.73%	72.41%	8.2	0.747	0.645 - 0.832
ΔSUVmax	93.94%	48.28%	1.9	0.732	0.629 - 0.820
ΔSUVmean	84.85%	56.90%	1.3	0.738	0.636 - 0.825

CI, confidence interval; SUVmax, maximum standard uptake value; SUVmean, mean standard uptake value; eSUVmax, early SUVmax; eSUVmean, early SUVmean; dSUVmax, delayed SUVmax; dSUVmean, delayed SUVmean; PD-L1, programmed death ligand 1.

### Correlations Between TILs and MPs on DTP FDG PET

**Figure 3:**
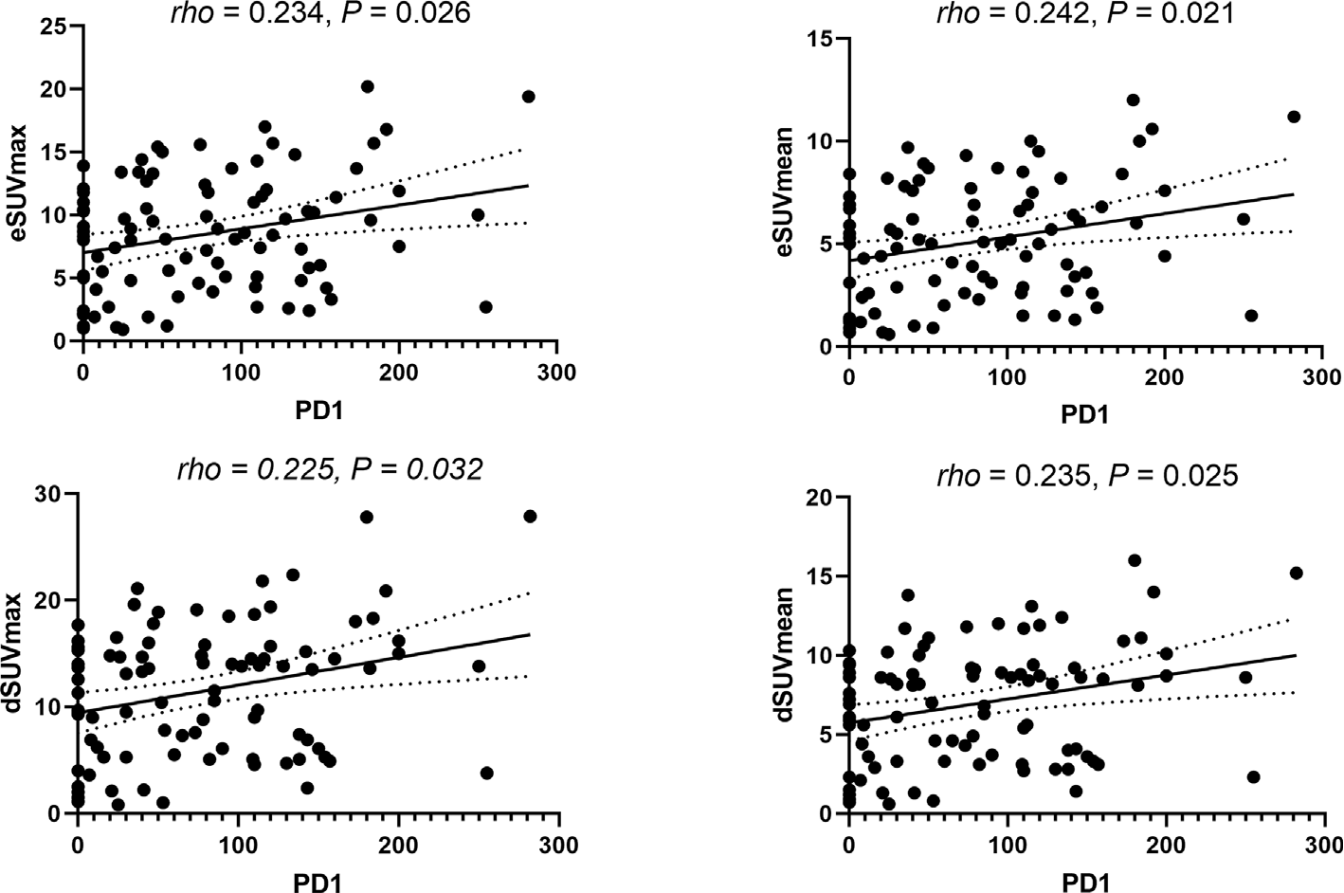
Correlations between metabolic parameters and PD-1 expression.

Spearman’s correlation coefficients revealed poor correlations between TILs and MPs. Specifically, CD8+ TILs in NSCLC were weakly correlated with dSUVmean (rho = 0.212, *p* = 0.044) and ΔSUVmean (rho = 0.209, *p* = 0.047). Similarly, weak correlations were found between PD-1 TILs and eSUVmax (rho = 0.234, *p* = 0.026), eSUVmean (rho = 0.242, *p* = 0.021), dSUVmax (rho = 0.225, *p* = 0.032) and dSUVmean (rho = 0.235, *p* = 0.025), respectively (**Figure 3**).

### Correlations Between Different TMITs and MPs on DTP FDG PET

**Figure 4:**
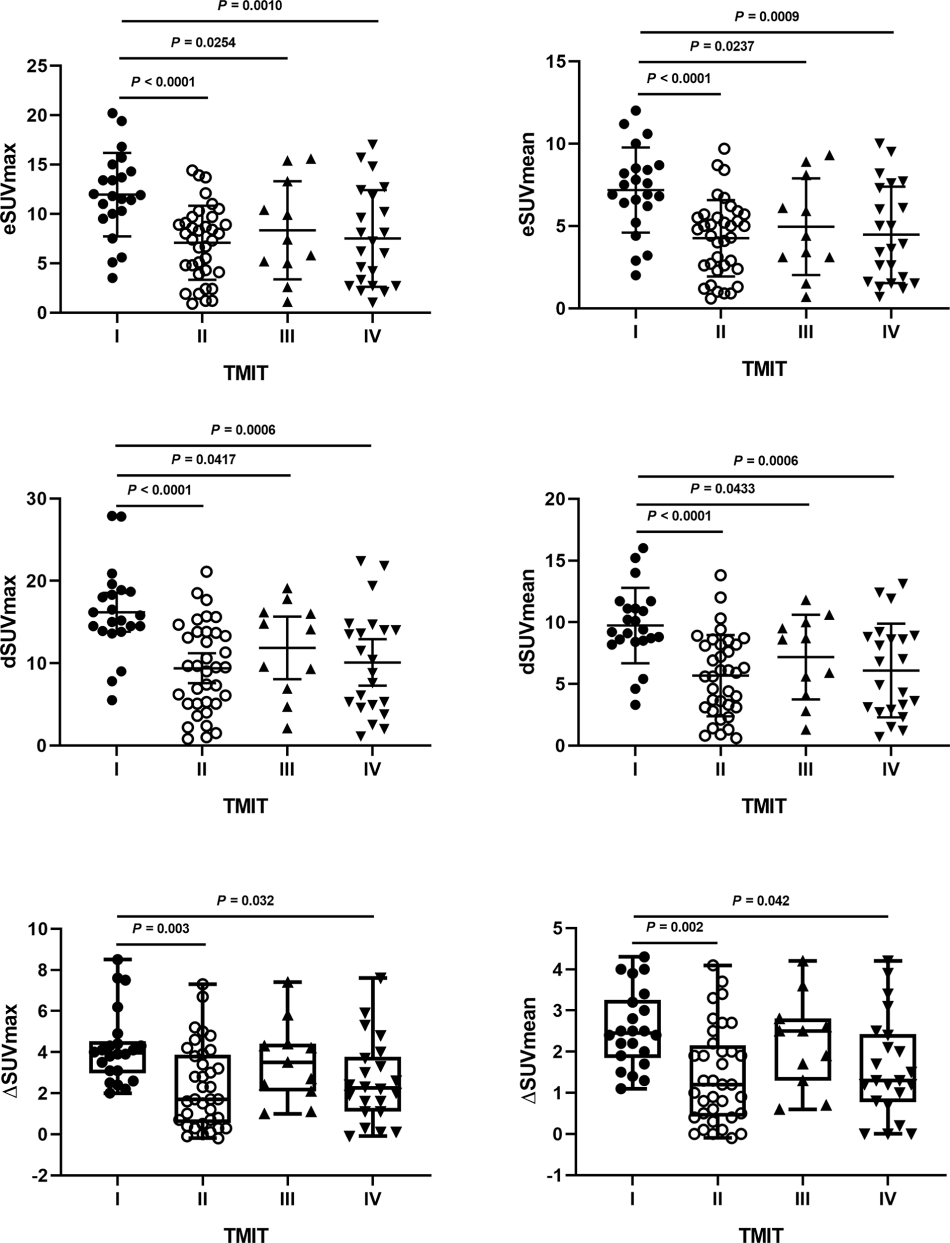
The differences of metabolic parameters (MPs) according to different tumor microenvironment immune types (TMITs). The MPs were significantly higher in TMIT I tumors than other immune types.

**Figure 5:**
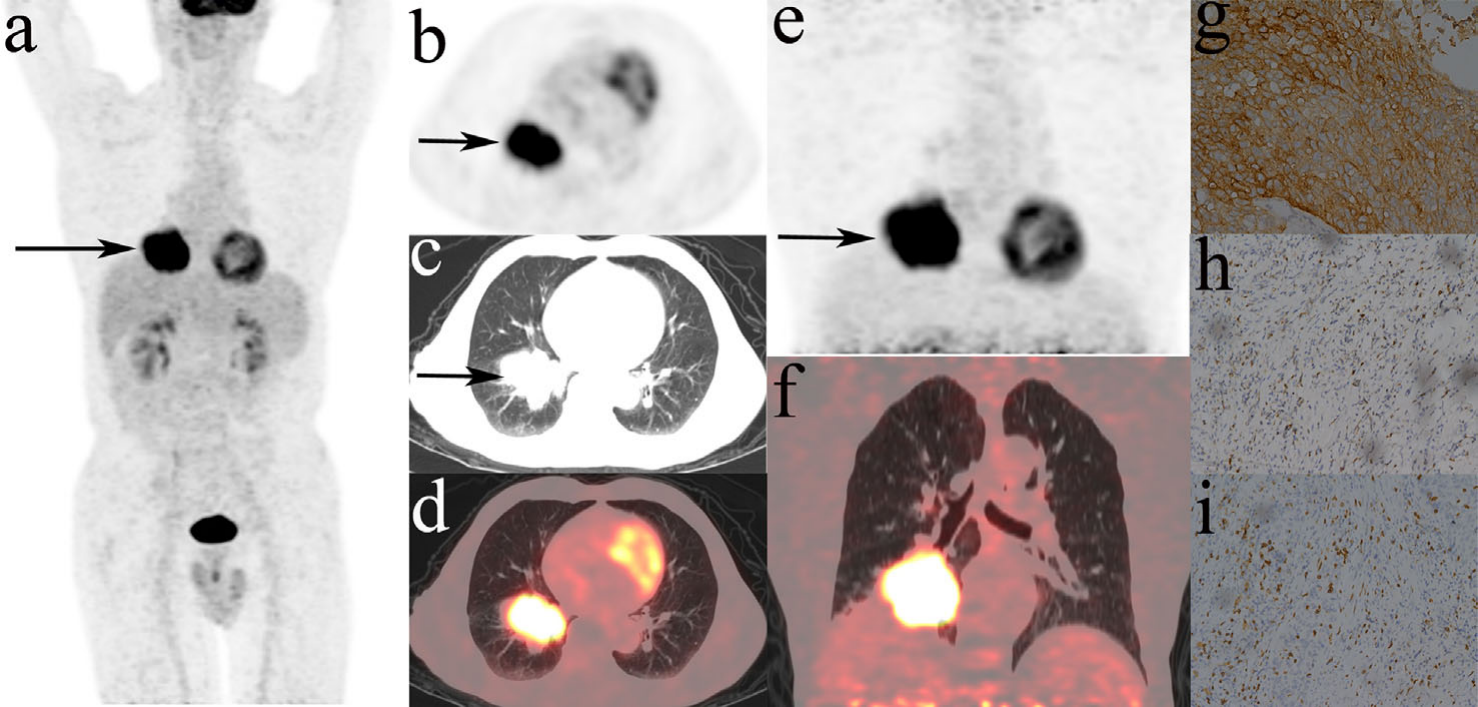
Representative DTP ^18^F-FDG PET/CT imagings of a 71y male patients, defined as TMIT I tumor. **(A–D)**: early images, **(E, F)**: delayed images, **(A)**: MIP figure, **(B–D)**: PET, lung window, PET/CT fusion image. A mass was in the lower lobe of right lung (arrow) with markedly increased radioactivity, eSUVmax: 20.3, eSUVmean: 12.0, eMTV: 52.9 cm^3^, eTLG: 634.8 g, dSUVmax: 27.7, dSUVmean: 16.0, dMTV: 49.16 cm^3^, dTLG: 786.56 g. The surgical pathology: moderately differentiated squamous cell carcinoma. **(G)** high PD-L1 expression. **(H)** PD-1 TIL high density. **(I)** CD8^+^ TIL high density.

**Figure 6:**
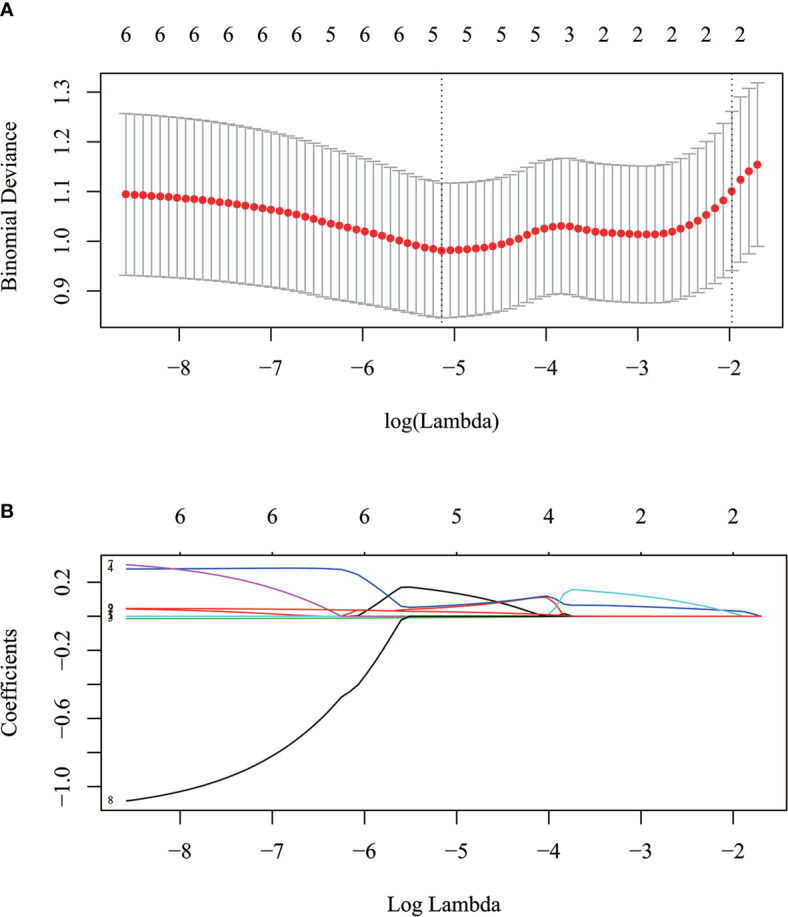
The selection of optimal MPs using the LASSO algorithm. **(A)** The optimal tuning parameter (Lambda) in the LASSO model was selected using 10-fold cross-validation at the minimum of lambda. **(B)** LASSO coefficient profiles of the 9 parameters. According to the 10-fold cross-validation in **(A)**, Five parameters with non-zero coefficients were included for metabolic signature construction.

**Figure 4** showed that most MPs were significantly different between TMIT I and other immune types (TMIT II, III, IV) respectively. Meanwhile, the least *p* value was shown in TMIT I vs II, increasing gradually in TIMT I vs IV and TIMT I vs III. Moreover, TMIT I tumors exhibited higher eSUVmax, eSUVmean, eTLG, dSUVmax, dSUVmean, dTLG, ΔSUVmax, ΔSUVmean and ΔTLG (*p* = 0.0001, < 0.0001, 0.0453, < 0.0001, <0.0001, 0.0231, 0.0009, 0.0009, 0.0144) than other types together (TMIT II+III+IV). **Figure 5** showed a representative patient with a TMIT-I tumor exhibited hypermetabolic tumors on DTP PET, characterized by high expression of PD-L1 and high density of PD-1, CD8^+^ TILs. Of the nine metabolic features above, eSUVmax, eSUVmean, eTLG, dSUVmax and ΔTLG were selected in the LASSO model with 10-fold cross-validation (**Figure 6**). The Meta-Sig (metabolic signature) was constructed as follows:

$$\begin{array}{lll} Meta-Sig=0.125626063\times eSUVmax+0.066810617\times eSUV—mean & \;-0.006982037\times eTLG+0.066701772\times dSUVmax & \;+0.024456035\times \Delta TLG-3.584450396. \end{array}$$

Meta-Sig had a higher AUC value than the stand-alone MP, although the differences were not statistically significant according to the Delong test. When compared to eSUVmax (AUC: 0.775, sensitivity: 77.27%, specificity: 73.91%) and dSUVmax (AUC: 0.788, sensitivity: 86.36%, specificity: 65.22%), Meta-Sig enhanced the performance to predict TMIT I tumors with higher sensitivity or specificity (AUC: 0.818, sensitivity: 86.36%, specificity: 73.91%) (**Table 3**).

**Table 3:** Performance of the combined model and other factors according to TMIT I tumors.

Factors	Sensitivity	Specificity	AUC	ΔAUC	95% CI	*Z*	*P*
Model	77.27%	82.61%	0.869				
dSUVmax	86.36%	65.22%	0.788	0.0814	0.0135 - 0.149	2.351	0.0187*
eSUVmax	77.27%	73.91%	0.775	0.0935	0.0159 - 0.171	2.361	0.0182*
Gender	68.18%	53.62%	0.609	0.260	0.140 - 0.380	4.250	< 0.0001*
Smoking	68.18%	68.12%	0.681	0.188	0.0840 - 0.291	3.551	0.0004*
Meta-Sig	86.36%	73.91%	0.818	0.0514	-0.0134 - 0.116	1.553	0.1203

DeLong method were used to compared the AUC of combined model with other factors.

*Statistically significant.

SUVmax, maximum standard uptake value; dSUVmax, delayed SUVmax; eSUVmax, early SUVmax; Meta-Sig, metabolic signature; CI, confidence interval; TMIT I: 3tumor microenvironment immune type I.

With multivariate logistic regression analysis (using backward stepwise elimination method), the combined model was constructed based on the clinical and metabolic information. The formula was as follows:

Model=1.490×Meta−Sig+2.435×smoking−0.840×stage−1.689×gender+1.883

**Figure 7:**
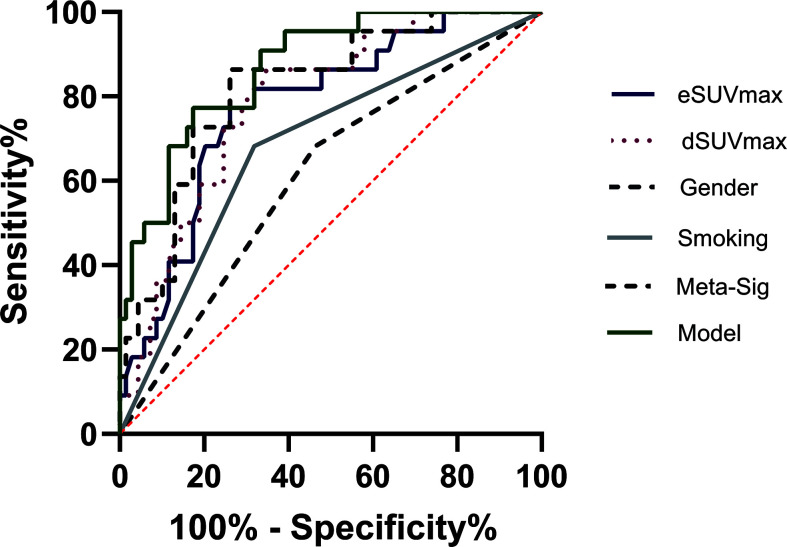
Representative image of receiver operating characteristic (ROC) curves for various factors in the analyses of TMIT I tumors. The combined model had the highest AUC than other factors.

**Figure 8:**
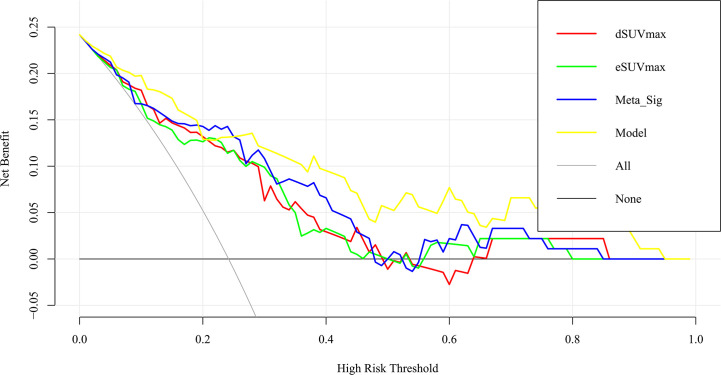
Decision curve analysis for the model and other factors. The y axis measures the net benefit. The x axis shows the threshold probability. The yellow line represents the combined model. The blue line represents the Meta-Sig only. The thin gray line represents the assumption that all patients were with TMIT I tumors. The black line represents the assumption that no patients have a TMIT I tumor.

The preferable model was assessed using ROC analyses and decision curve analysis (DCA). Compared to the combined model (AUC: 0.869, sensitivity: 77.27%, specificity: 82.61%), AUCs for eSUVmax, dSUVmax, gender, and smoking were relatively lower according to the Delong test (*p* = 0.0187, 0.0182, <0.0001, 0.0004) (**Table 3**; **Figure 7**). DCA for the combined model was shown in **Figure 8**. Using the combined model to predict TMIT I tumors added more benefit than eSUVmax or dSUVmax.

## Discussion

The past decade was marked by a revolution in the field of cancer treatment. Recently, antibody-based immunotherapy that modulates immune responses against tumors has been approved as first-line treatment option for selected advanced or metastatic lung cancer (20). However, the response of NSCLC patients to immunotherapy is affected by the TIME. Notably, patients with TMIT I tumors, regarded as immunologically ‘hot’, are most likely to benefit from ICI therapy. Our study provided a new insight into the underlying correlation between TIME types and DTP FDG PET imaging. To our knowledge, this is the first study to identify TMIT I type using DTP FDG PET scan in pretreated NSCLC patients.

In general, TMIT I tumors are characterized by somatic tumor mutations, PD-L1/PD-1 expression, and CD8^+^ TILs. In a previous report, TMIT I tumors were found to harbor significantly more somatic tumor mutations (6), therefore presenting more neoantigens (4). Endogenous CD8^+^ T cells can recognize these neoantigens, increase the TILs density, and trigger an immune response by the host (21). Meanwhile, glucose transporter 1 is reported to upregulate in these activated CD8^+^ T cells, leading to increased glucose uptake (8). Furthermore, during therapeutic PD-1 blockade, pre-treatment samples obtained from responders exhibited higher CD8^+^ cell densities compared to those from non-responders (22). According to results in our study, tumors with high infiltration of CD8^+^ T cells had a tendency for high FDG uptake in comparison with the TILs^-^ tumors. It suggested that FDG PET could serve as a non-invasive tool to assess the tumor microenvironment, and might help to identify responders ahead of treatment. However, CD8+ TILs alone had low correlations (*p* = 0.044, 0.047) with MPs in our work, proving the necessity of simultaneously interpreting multiple immune biomarkers within the complex system of tumor immune microenvironment. Indeed, there was a metabolic competition between tumors and immune cells. The high levels of glycolysis within tumor cells consumed extracellular glucose, which in turn impaired the glycolysis in T cells (23). We hypothesized that the FDG uptake of TMIT I tumors depends primarily on PD-L1+ tumor cells rather than CD8^+^ T cells. Chang et al. (24) reported that PD-L1 expression maintained Akt/mTOR signaling, which in turn promoted metabolic pathway through the translation of glycolysis enzymes. Lopci et al. (9) first investigated the correlation between PD-L1 expression and FDG uptake in NSCLC. Although no correlation was found in this particular study, the negative results may arise from the small size of studied patients. Later, Takada et al. (10) found a positive correlation between FDG uptake with PD‐L1 expression in a larger group of patients. In addition to PD-L1 expression and CD8^+^ TILs, PD-1 expression is also a biomarker of ICIs treatment. PD-1 expression was significantly higher in TMIT I tumors than type II and III, probably because PD-1 is of upregulated in CD8+ TILs, where the binding of PD-1 and its ligand PD-L1 can inhibit a cytotoxic T-cell response.

CD8+ TILs and the tumor immune microenvironment may also affect patients’ responses to immunotherapy, since not all patients with positive PD-L1 expression respond well. We hereby categorized the tumor immune microenvironment into four subgroups to assist the stratification of NSCLC patients. We strived to identify responders from the perspective of different tumor immune types, and for the first time established the correlation between dynamic glucose metabolism and TMIT-I tumors. One remarkable finding in the present study was that TMIT I tumors present higher MP values than other types (TMIT II, III, IV), and the smallest *p* value was shown between TMIT I vs II. Contrary to TMIT I tumors, TMIT II tumors, regarded as “cold” tumors, lack tumors cells expressing PD-L1 and CD8^+^ T cells. While TMIT I tumors are most likely to benefit from single-agent anti-PD-1/L1 blockade, TMIT II tumors had significantly worse prognosis due to the lack of detectable immune responses (4). The semi-quantification of MPs on DTP FDG PET was a decision support methodology for the complex clinical decisions to differentiate TMIT I and II tumors. Compared to TMIT I vs II, the *p* values increased gradually in TMIT I vs IV. TMIT IV tumors are enriched with CD8^+^ TILs. However, the lower uptake FDG may be attributed to PD-L1 negativity. Interestingly, TMIT III tumors also showed relatively lower FDG uptake than TMIT I, and the *p* value increased even further in TMIT I vs III. Since TMIT III tumors were also characterized by PD-L1 positivity like TMIT I tumors, CD8^+^ T cells have less effect on the FDG uptake of TMIT III tumors. It should be noted that only a low proportion of tumors in this study belonged to TMIT III (12.1%, 11/91). A similarly low occurrence of TMIT III was also observed in melanoma. (2%) (25). Nevertheless, the presence of TMIT III tumors indicates that not all patients with PD-L1 positive expression respond well to the ICIs. Our work demonstrated the importance of highlighting this subset of patients as probable non-responders to immunotherapy. The identification of different tumor immune types is meaningful from the perspective of clinical practice, since using the same strategy for all patients will be inefficient, costly, and unreasonable (26).

For the identification of TMIT I tumors, DTP FDG PET not only provided a non-invasive work-up, but also improved the capability for pre-selecting patients that are likely responsive to ICIs. Cancer cells continuously uptake ^18^F-FDG and trap them intracellularly in the form of ^18^F-FDG-6-phosphate (27). In contrast, in benign tissue, the uptake of ^18^F-FDG decreases or plateaued after reaching a maximum within 30 min of FDG administration. Although it is generally accepted that FDG-PET/CT images obtained during the delayed phase reflect the dynamics of tumor glucose metabolism, our study revealed that eSUVmax and dSUVmax had similarly unsatisfactory performances for the assessment of TMIT I tumors since the sensitivity of eSUVmax (77.27%) and specificity of dSUVmax (65.22%) were relatively low when compared with each other. The application of DTP FDG PET provided a new perspective for the assessment of tumor immune microenvironment, and can make up the deficiencies of a single MP. Compared with the MP at a single time point, DTP PET exhibited improved sensitivity (Meta-Sig vs eSUVmax: 86.36% vs 77.27%) and specificity (Meta-Sig vs dSUVmax: 73.91% vs 65.22%). Therefore, DTP FDG PET might reduce the false positive rates on early scan and false negative rates on delayed scan, which would help to facilitate accurate treatment strategies and reduce unnecessary medical cost. Furthermore, the combined model achieved a performance for the identification of TMIT I tumors with AUC of 0.869, better than that of Meta-Sig (0.818). As confirmed by DCA, the combined model had a better clinical usefulness than Meta-Sig at a wide range of threshold probability (except the range of 20%~25%).

This present study also has a couple of limitations. First, this study was a single institutional retrospective study with a limit number of subjects. Second, further understanding of the complex and volatile TIME is still needed because TIME consists of various cells types, which display metabolic interactions in the tumor microenvironment. Third, as the analysis of histology slices was performed with a semi-quantitative method in the present study, some clustering analyses are needed to make it more quantitative. The clustering analysis may allocate the cohort into distinct and discrete subgroups, and clearly delineate patients who are suitable for immunotherapy. Fourth, none of the patients in the retrospective cohort received immunotherapy within one month of the 18F-FDG PET/CT scan. Therefore, it is unknown whether the patients with significantly changed MPs could benefit from immunotherapy. A recent study on 89 patients with advanced or recurrent NSCLC showed that patients with higher baseline SUVmax values had higher response rate to immunotherapy than those with lower baseline SUVmax values (28). Future studies are warranted to enroll patients that receive subsequent 18F-FDG PET/CT and immunotherapy within one month of each other, and to evaluate whether the patients with significantly changed MPs could benefit from immunotherapy.

## Conclusion

High glucose metabolism on DTP ^18^F-FDG PET is relevant to TMIT-I tumors, and the Meta-Sig and combined model based on clinical and metabolic information could improve the performance of identifying patients who may respond to ICIs treatment.

## Data Availability Statement

The original contributions presented in the study are included in the article/supplementary material. Further inquiries can be directed to the corresponding authors.

## Ethics Statement

All procedures performed in studies involving human participants were approved by the Institutional Review Board of Tongji Hospital, Tongji Medical College, Huazhong University of Science and Technology (TJ-IRB 20181202) and in accordance with the principles of the 1964 Declaration of Helsinki and its later amendments or comparable ethical standards. This article does not describe any studies with animals performed by any of the authors.

## Author Contributions

XZ, JZ and SZ contributed to the conception and design of the study. JZ, SC, DK, DL, LC and CL carried out the research. JZ, SZ, JY and XZ performed the data analysis. JZ wrote the first draft of the manuscript. XZ, SZ, DK and JY made the comments. XZ and SZ critically reviewed and revised the manuscript. All authors contributed to the article and approved the submitted version.

## Funding

This work was supported by the National Natural Science Foundation of China (81671718, 81873903, 91959119), and Natural Science Foundation of Hubei Province of China (2016CFB687).

## Conflict of Interest

The authors declare that the research was conducted in the absence of any commercial or financial relationships that could be construed as a potential conflict of interest.
